# Modeling Diabetic Neuropathy: Injury Biomarkers for Early Detection Through Neuronal and Glial In Vitro Models

**DOI:** 10.3390/ijms27188234

**Published:** 2026-09-16

**Authors:** Luisa Muratori, Simona Rando, Stefania Raimondo

**Affiliations:** 1 Department of Clinical and Biological Sciences, University of Torino, 10043 Orbassano, Italy; luisa.muratori@unito.it (L.M.); simona.rando@unito.it (S.R.); 2 Neuroscience Institute Cavalieri Ottolenghi (NICO), University of Torino, 10043 Orbassano, Italy

**Keywords:** diabetes, nerve damage, biomarker, diabetic peripheral neuropathy, hyperglycemia, hypoxia, neuronal injury

## Abstract

Diabetes mellitus (DM) is a rapidly expanding global health burden and a leading cause of chronic complications. Among these, diabetic peripheral neuropathy (DN) is one of the most prevalent and disabling, affecting up to half of individuals with long-standing diabetes and involving both somatic and autonomic nerve fibers. DN is characterized by an insidious onset: axonal and glial damage begins during subclinical phases and is frequently detected only when irreversible injury has occurred, as conventional neurological examinations and nerve conduction studies lack sensitivity for detecting early-stage disease. DN pathogenesis is multifactorial and primarily driven by chronic hyperglycemia, which triggers a network of converging pathogenic mechanisms. This narrative review aims to explore the evolving landscape of DN, with a particular focus on the study of neuronal injury biomarkers in the early detection of diabetic nerve damage. In addition, it highlights the relevance of in vitro neuronal and glial models as complementary experimental platforms to investigate the molecular and cellular mechanisms underlying DN and to support the identification and validation of candidate biomarkers. Moreover, these in vitro systems provide a strategic tool for examining the impact of circulating factors on axonal and glial integrity, thereby facilitating the discovery and preliminary assessment of potential blood-based biomarkers.

## 1. Introduction

Diabetic peripheral neuropathy (DN) is a chronic, length-dependent axonopathy characterized by the progressive degeneration of sensory, motor, and autonomic nerve fibers. Nerve fiber degeneration typically begins in the longest peripheral axons, initially affecting small unmyelinated C-fibers and thinly myelinated Aδ fibers before extending to large myelinated fibers responsible for proprioception and motor function [1,2]. This distal-to-proximal pattern reflects the high metabolic demand and limited regenerative capacity of long peripheral axons under chronic diabetic conditions. One of the hallmarks of DN is its prolonged subclinical phase. Axonal degeneration, Schwann cell (SC) dysfunction, demyelination, microvascular impairment, and the progressive loss of neurotrophic support develop long before overt neurological symptoms become evident, resulting in a gradual decline in nerve structure and function that frequently remains undetected until irreversible damage has occurred, which limits the effectiveness of therapeutic intervention [3,4]. Although distal sensorimotor polyneuropathy represents the most common clinical manifestation [2,5,6], diabetic neurodegeneration is not restricted to somatic nerves but also involves autonomic compartments (Table 1). Autonomic neuropathy develops in a length-dependent manner, preferentially affecting the longest fibers, such as the vagus nerve, before progressively involving sympathetic and parasympathetic innervation of cardiovascular, gastrointestinal, genitourinary, sudomotor, and ocular systems [3,7,8,9,10]. Although the clinical manifestations differ according to the target organ, autonomic neuropathies may lead to life-threatening complications. Cardiac Autonomic Neuropathy (CAN) is associated with increased risk of sudden cardiac death, which makes it one of the most clinically relevant manifestations of diabetic neurodegeneration [3,7]. Together, these observations highlight that DN should be considered as a multifaceted neurodegenerative disorder in which common molecular mechanisms drive progressive nerve degeneration across both somatic and autonomic pathways.

Understanding the interconnected mechanisms leading to irreversible nerve damage is essential not only for elucidating disease pathophysiology but also for identifying reliable biomarkers of early nerve degeneration. In this context, advanced neuronal and glial in vitro models represent powerful translational platforms for reproducing disease-relevant mechanisms and investigating the molecular pathways regulating nerve degeneration and regeneration. Accordingly, this review focuses on the molecular mechanisms underlying nerve degeneration in DN and discusses how advanced in vitro neuronal and glial models can be exploited to unravel these mechanisms. Particular emphasis is placed on the use of in vitro platforms for biomarker discovery, an aspect that has received comparatively limited attention in previous reviews. By integrating disease-relevant molecular mechanisms with the experimental models available to reproduce them, we highlight how neuronal, SC, and multicellular platforms can support the identification, characterization, and functional validation of candidate biomarkers. This model-based perspective may help bridge the gap between mechanistic knowledge and biomarker development, enabling the early detection of DN before the occurrence of clinical manifestations.

## 2. Data Sources

The literature search followed a narrative approach and focused on studies investigating the molecular mechanisms, biomarkers, and experimental models of diabetic neuropathy. Searches combined the terms “diabetic neuropathy” OR “diabetic peripheral neuropathy” with “biomarker”, “neurofilament”, “GFAP”, “RAGE”, “Schwann cell”, “dorsal root ganglion”, “in vitro”, “organoid”, “microfluidic”, “high glucose”, and “hypoxia” in PubMed, Embase, Scopus, Web of Science, and Google Scholar, with coverage from database inception to June 2026. The selection was purposive and concept-driven, and no formal risk-of-bias assessment or quantitative synthesis was undertaken.

## 3. Molecular Mechanisms of DN

**Figure 1:**
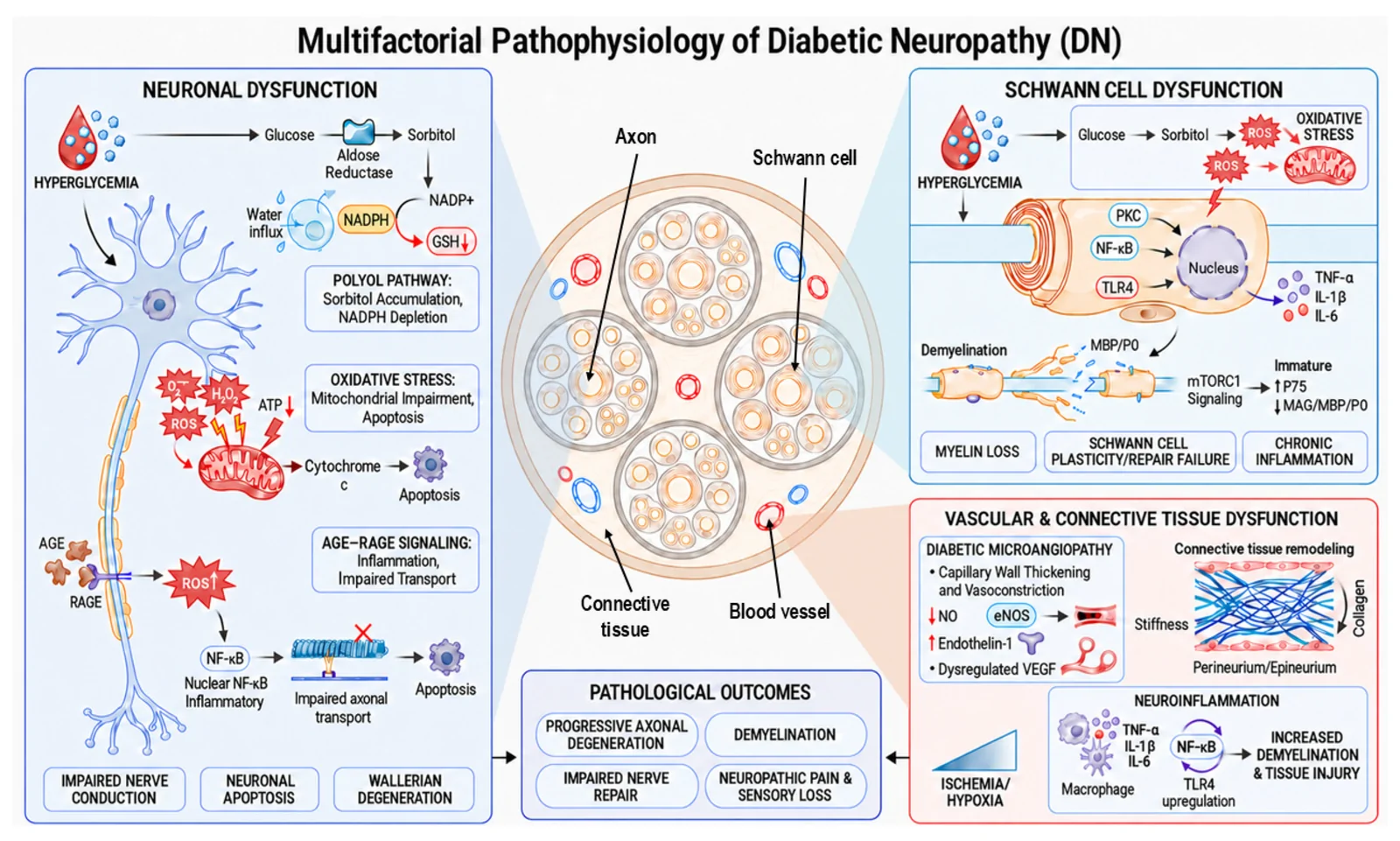
Schematic representation of multifactorial pathophysiology of DN. Hyperglycemia induces neuronal dysfunction (**left**) through polyol-pathway activation, oxidative stress and AGE–RAGE signaling, leading to impaired nerve conduction, apoptosis and degeneration. Through diabetic hyperglycemia, Schwann cell dysfunction (**upper right**) results in myelin loss, cellular plasticity failure and impaired repair mechanisms with establishment of chronic inflammation. Vascular and connective tissue (**lower right**) contributes to the onset of the disease by diabetic microangiopathy, with capillary wall thickening and vasoconstriction, tissue remodeling and persistent resident macrophage activation. Overall, these molecular mechanisms will lead to progressive axonal damage, demyelination, impaired nerve repair and, eventually, to neuropathic pain and sensory loss. Graphical representation was created with the assistance of ChatGPT-5 (OpenAI, 2026) and scientifically reviewed by the authors.

The pathophysiology of DN is multifactorial, involving a complex interplay of metabolic disturbances, oxidative stress, vascular dysfunction, chronic inflammation, and impaired neuronal repair mechanisms [11,12]. Different cellular components are affected by various dysfunctional pathways, with neuronal and glial compartments being the main characters involved [13] (Figure 1).

### 3.1. Neurons

Chronic hyperglycemia, at the core of DN, drives neuronal injury, initiating a cascade of damaging biochemical pathways. One of the earliest is the activation of the polyol pathway, where excess glucose is converted to sorbitol by the enzyme aldose reductase [14]. Sorbitol is osmotically active and poorly diffusible across cell membranes. Its buildup causes water influx, leading to cell swelling and dysfunction. Moreover, this reaction consumes NADPH, a vital cofactor for regenerating glutathione, the cell’s primary antioxidant. As NADPH is depleted, oxidative stress increases, damaging cellular components and impairing nerve conduction [15,16].

Hyperglycemia also promotes the formation of advanced glycation end-products (AGEs). AGEs are harmful compounds that form when proteins, lipids, or nucleic acids become non-enzymatically but chemically glycated with reducing sugars such as glucose. These compounds bind to their receptor, the receptor for advanced glycation end-products (RAGE), on neurons and endothelial cells, activating intracellular signaling cascades that increase reactive oxygen species (ROS) production and trigger inflammatory responses. AGEs interfere with axonal transport and mitochondrial function, leading to neuronal apoptosis and the loss of nerve integrity [17]. RAGE biology is further complicated by alternative splicing. Full-length membrane RAGE transduces ligand-dependent signals, whereas endogenous secretory RAGE (esRAGE) and other soluble RAGE forms lack the transmembrane domain and can bind circulating ligands [18,19,20]. Soluble RAGE may therefore act as a decoy receptor by sequestering AGEs and other RAGE ligands, while membrane RAGE can amplify oxidative stress, NF-κB-dependent inflammation, and vascular or neural injury. The balance between these forms, membrane RAGE and esRAGE, could consequently be relevant to DN, because altered isoform expression influences the intensity of AGE–RAGE signaling in neurons, SCs, and endoneurial vascular cells.

Moreover, oxidative stress, often seen as a consequence of increased blood glucose concentration, plays a central role in DN. Mitochondria, the primary source of ROS under hyperglycemic conditions, become dysfunctional, which impairs ATP production and increases oxidative damage [21]. Impaired mitochondrial electron transport chain leads to ROS production, increasing proton leakage and altering mitochondrial membrane potential. The release of cytochrome c from damaged mitochondria activates caspase-dependent apoptosis, further contributing to neuronal loss [22]. These changes disrupt axonal transport and promote Wallerian degeneration. Small unmyelinated C-fibers and thinly myelinated Aδ fibers are particularly vulnerable, contributing to early sensory deficits and neuropathic pain [23].

### 3.2. Schwann Cells

SCs are essential for peripheral nerve myelination, axonal maintenance, and the guidance of nerve repair following injury [24,25,26]. Consequently, SC dysfunction and demyelination are key contributors to the structural nerve damage observed in DN. In DM, metabolic and inflammatory stressors impair SC function, thereby exacerbating peripheral nerve damage [27,28].

Similarly to neurons, the polyol pathway is up-regulated in SCs, where aldose reductase converts excess glucose into sorbitol, consuming NADPH and amplifying oxidative stress [29]; mitochondrial dysfunction follows, with an impaired electron transport chain that generates excess ROS and triggers cytochrome c release, mirroring neuronal apoptosis [30]; AGE-RAGE signaling also operates in SCs, further elevating ROS and inflammatory cascades [30]; chronic activation of PKC, NF-κB and TLR4 pathways sustains the production of TNF-α, IL-1β and IL-6, driving demyelination and a shift toward an immature, repair phenotype marked by increased P75 and decreased MAG/MBP/P0 expression [30]; finally, dysregulated mTORC1 signaling under hyperglycemia promotes SC plasticity that favors dedifferentiation and impairs remyelination [29]. Together, these convergent pathways erode SC myelin maintenance, axonal support, and regenerative capacity, which highlights the central role of SC plasticity in DN [28].

### 3.3. Vascular and Connective Tissue Components

Vascular dysfunction compounds the problem. An increased blood glucose concentration can damage small capillaries, resulting in diabetic microangiopathy. Endothelial cells exposed to high glucose levels produce less nitric oxide due to the impaired activity of endothelial nitric oxide synthase (eNOS), which results in vasoconstriction and reduced perfusion of peripheral nerves [31,32]. At the same time, levels of endothelin-1, a potent vasoconstrictor, are elevated, which further limits blood flow [33,34]. Thickening of the capillary basement membrane reduces oxygen diffusion, contributing to nerve ischemia and hypoxia. Vascular endothelial growth factor (VEGF), which normally promotes angiogenesis, is dysregulated, which leads to abnormal vessel formation and impaired nerve repair [35].

Neuroinflammation is another major contributor to DN, particularly through activation of the NF-κB pathway, a transcription factor that promotes the expression of inflammatory cytokines such as TNF-α, IL-1β, and IL-6. These cytokines activate resident macrophages, which leads to chronic inflammation and demyelination [36]. Toll-like receptor 4 (TLR4) is up-regulated in SCs and immune cells, which amplifies the inflammatory response. The degradation of IκBα, an inhibitor of NF-κB, allows sustained activation of this pathway, perpetuating tissue damage [37]. Additionally, connective tissue structures, including the perineurium and epineurium, also undergo remodeling in DN. Fibrosis and collagen deposition increase mechanical stiffness and reduce nerve elasticity and gliding [38,39].

Overall, DN results from a complex interplay of metabolic, inflammatory, and vascular insults. Chronic hyperglycemia drives oxidative stress, mitochondrial dysfunction, and persistent inflammation, while vascular impairment and insulin resistance further compromise neuronal survival and repair. The dysregulation of key pathways, including the polyol and hexosamine pathways and NF-κB and PI3K/Akt signaling, contributes to the progressive degeneration of peripheral nerves [36,40]. Elucidating these mechanisms is critical for the development of targeted therapies, and timely diagnosis combined with personalized interventions remains essential to improve patient outcomes.

## 4. Biomarkers for Early Detection of Neuronal Injury

Early detection of DN remains a critical unmet need because once neuronal loss and axonal degeneration become clinically apparent, nerve damage is often irreversible and associated with severe complications. Traditional clinical examinations and nerve conduction studies lack sensitivity for subclinical disease, which has prompted a shift toward blood-based biomarkers capable of detecting neuronal injury before overt symptoms emerge.

**Figure 2:**
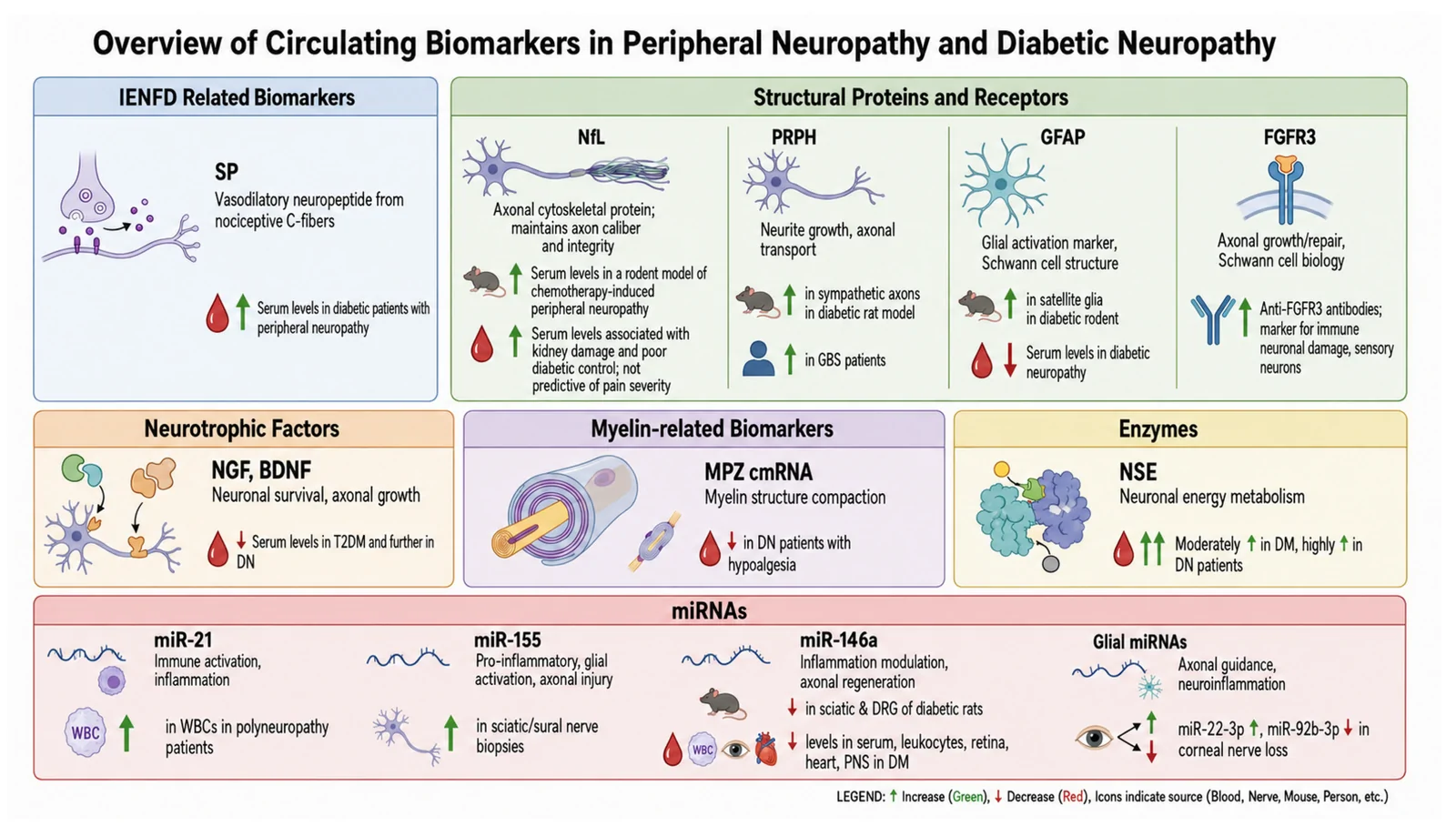
Schematic overview of circulating biomarkers of axonal and glial damage in diabetic neuropathy. Biomarkers are grouped according to the text: IENFD-related, structural proteins, neurotrophic factors, myelin-related, enzymes, and miRNAs. Graphical representation was created with the assistance of ChatGPT-5 (OpenAI, 2026) and scientifically reviewed by the authors.

Several studies illustrate how in vitro models can move candidate biomarkers from mechanistic observation toward functional evaluation, and the aim of this work is to argue that the discovery of novel, reliable biomarkers for DN depends on the rigorous use of well-designed in vitro models [41]. The following examples of experimental evidence, paired with clinical studies, show that in vitro systems can do more than reproduce diabetic injury: they enable identification of the cellular sources and temporal behaviors of candidate biomarkers, connecting their changes to a measurable axonal or glial phenotype to finally test whether the signal is modifiable. The resulting biomarkers should be regarded as mechanistically supported candidates requiring subsequent analytical, biological, and clinical validation rather than as clinically established diagnostic markers. Beyond the anticipatory function for irreversible diagnosis, this model-based perspective can further enhance risk stratification, enabling clinicians to identify high-risk patients and initiate timely interventions. The following sections describe selected biomarkers and discuss their potential roles in the early diagnosis of DN, as reported in Figure 2.

### 4.1. Intraepidermal Nerve Fiber Density (IENFD)-Related Biomarkers

Intraepidermal Nerve Fiber Density (IENFD) is a skin biopsy test that measures the number of small nerve fibers in the skin’s epidermis to diagnose small fiber neuropathy. Significant changes in IENFD have been demonstrated in DN patients, related to the duration of diabetes, particularly a shortening of IENFD from proximal to distal sites and a significant loss of fibers at the distal site of the lower limb [42].

Calcitonin gene-related peptide (CGRP) and substance P (SP) are neuropeptides that are released from nociceptive C-fibers. As these fibers deteriorate, their ability to synthesize and release neuropeptides diminishes. Both being vasodilator neuropeptides, CGRP is more potent, inducing a long-lasting increase in superficial skin blood flow, whereas SP induces only a brief vasodilation but a significant plasma extravasation [43].

No clinical evidence suggesting changes in serum levels of CGRP was found in the literature; serum levels of SP appear to be decreased in diabetic patients with peripheral neuropathy but do not appear to be correlated with the presence of retinopathy [44]. Conversely, keratopathy, involving pain sensation and corneal changes, appears to be linked with SP serum levels. This was confirmed by analysis of the tear film in type 1 diabetes (T1DM) patients with peripheral neuropathy, which showed a decreased concentration of SP associated with corneal changes [45].

### 4.2. Structural Proteins

Neurofilament Light Chain (NfL) is a key structural protein found primarily in neurons, specifically within the cytoskeleton of axons. It plays a critical role in maintaining axonal caliber and neuronal integrity. When axons are damaged, NfL is released into the extracellular space and enters into circulation, causing increased serum levels [46]. Experimental animal models showed correlations between axonopathy and serum NfL levels [47], and prevalent DN has been demonstrated in patients with early T1DM and T2DM [48,49]. Moreover, serum NfL levels were found to be associated with kidney damage and poor diabetic control but without predictive function for pain severity [50].

Recent studies have highlighted peripherin (PRPH), a class III intermediate filament protein, as a promising biomarker for axonal damage in the peripheral nervous system [51]. Its expression is primarily localized in the PNS, particularly in motor neurons and primary sensory neurons, where it is believed to play a crucial role in neurite growth and stability, as well as in axonal transport during myelination. Regarding DN, experimental work in streptozotocin-induced type 1 diabetic rats shows an increase in PRPH protein in sympathetic axons innervating plantar metatarsal arteries, indicating tissue-level up-regulation in diabetes-related nerve damage [52]. Likewise, a mouse model of T2DM reported a rise in the proportion of PRPH+ small-diameter neurons in the lumbar dorsal root ganglion (DRG), which reflects altered neuronal composition rather than circulating concentrations [53]. Clinically, longitudinal serum measurements in cohorts of Guillain–Barré syndrome (GBS), chronic inflammatory demyelinating polyneuropathy, multiple sclerosis, and healthy controls showed that peak PRPH concentrations were significantly higher in GBS than in any other group [51]. Together, these findings suggest that PRPH expression is altered in diabetic nerve pathology, highlighting its possible use as a biomarker for DN.

GFAP, or glial fibrillary acidic protein, is an intermediate filament enriched in non-myelinating SCs; its up-regulation reflects cytoskeletal remodeling and glial activation in response to peripheral nerve injury, inflammation, or Remak bundle disruption [54,55]. In rodent studies of diabetes-induced nerve injury, satellite glial cells surrounding DRG neurons exhibit marked GFAP elevation, which indicates activation of these glial cells in response to hyperglycemic stress [56]. However, clinically, serum GFAP concentrations are reduced in individuals with DN compared with controls, which demonstrates its clinical relevance as a glial-derived biomarker [57]. Several factors may account for these divergent findings. For instance, GFAP elevation is well documented in inflammatory demyelinating neuropathies, but diabetic neuropathy may involve distinct pathogenic pathways that suppress systemic GFAP release. Moreover, animal models often capture acute or subacute glial activation, while human patients are usually examined at chronic stages when glial turnover declines and GFAP production may be exhausted, which can result in reduced serum concentrations.

Lastly, antibodies against fibroblast growth factor receptor 3 (FGFR3), which have been reported to delineate a distinct subgroup of immune-mediated sensory neuropathies, are also considered in this review as emerging candidate biomarkers. Their potential relevance lies in their ability to identify specific pathogenic mechanisms and patient subgroups, thereby contributing to a more refined biomarker-based stratification of DN [58].

### 4.3. Neurotrophic Factors

DM reduces the availability of and responsiveness to neurotrophic factors like nerve growth factor (NGF) and brain-derived neurotrophic factor (BDNF), which are essential for nerve survival and repair [59]. This neurotrophic deficit impairs axonal regeneration and may delay functional recovery even after glycemic control is improved. Clinical studies consistently demonstrate that circulating NGF and BDNF are markedly lower in patients with T2DM and are further reduced in those who develop DN. In a cohort of 83 T2DM patients and 65 DN patients, serum NGF and BDNF levels showed the greatest decline in the DN group, correlating with longer disease duration, higher fasting C-peptide, elevated HbA1c, and increased 24 h urinary microalbumin excretion [60].

### 4.4. Myelin-Related Biomarkers

In the context of demyelinating neuropathy, the injury or demyelination of large, myelinated fibers leads to reduced expression of myelin-related genes, which can be reflected in impaired circulating mRNA (cmRNA) levels. Emerging clinical data support the use of circulating myelin protein zero (MPZ) mRNA as a potential biomarker for large-fiber involvement in diabetic neuropathy.

Experimental mouse models involving genetic modification in the MPZ gene are used to mimic Charcot–Marie–Tooth disease type 1B to study demyelinating neuropathies [61]. Clinically, decreased MPZ cmRNA is associated with hypoalgesia in DN patients, preceding deficits in thermal and mechanical detection by 24 months. This could be a valuable tool for the early diagnosis of nerve fiber loss before Quantitative Sensory Testing or Nerve Conduction Velocity measurements show significant changes [62].

### 4.5. Enzymes

Neuron-Specific Enolase (NSE) is a glycolytic enzyme highly expressed in neurons and neuroendocrine cells, where it plays a role in energy metabolism [63,64]. In the context of neuronal injury, including DN, NSE is released into the extracellular space and becomes detectable in peripheral blood [64].

Serum NSE levels were shown to be moderately elevated in diabetic patients and highly elevated in DN patients when compared with DM patients and healthy individuals. Interestingly, the rise in NSE is independent of blood glucose levels or diabetes duration, which suggests that it is more directly related to nerve health than to general metabolic control [65]. Moreover, these findings are confirmed by prospective studies in patients with T2DM with and without DN [66,67].

### 4.6. MicroRNA or MiRnome Profiling

Growing research highlights the pivotal role of microRNAs (miRNAs) in the development of DN. These small, non-coding RNA molecules, typically 18 to 24 nucleotides in length, regulate gene expression by binding to mRNAs, which leads to translational repression or mRNA degradation [68]. Once matured, miRNAs can be secreted into the bloodstream and various body fluids. Their stability is remarkably high, thanks to protective carriers such as RNA-binding proteins and lipid-based vesicles, including microvesicles, exosomes, apoptotic bodies, and high-density lipoproteins [69].

Recent advances in the study of DN have underscored the critical involvement of miRNAs in its pathogenesis [70]. Relevant examples include the following miRNAs: miR-21, a key regulator of immune processes, was found to be significantly elevated in white blood cells from patients with polyneuropathies of various etiologies [71,72]; miR-155, a prototypic pro-inflammatory miRNA, drives glial activation and axonal injury in DN, and, consistent with experimental evidence, its expression is markedly elevated in sciatic nerve and sural nerve biopsies from patients with painful neuropathy [72,73,74]; and miR-146a, one of the most extensively studied miRNAs, has been associated with pain conditions in murine models, inflammatory degeneration, nerve regeneration following axonal injury, and distal axonal growth. Moreover, in a diabetic state, reduced expression has been observed in the serum, leukocytes, retina, heart, and peripheral nervous system [75,76,77,78,79]. Additionally, glial-related miRNAs like miR-22-3p (up-regulated) and miR-92b-3p (down-regulated) are shown to be relevant in patients with corneal nerve loss, which reflects altered axonal guidance and neuroinflammation [80].

Although individual miRNAs such as miR-21, miR-155 and miR-146a have demonstrated associations with pain modulation, immune response, and vascular dysfunction, their expression profiles often reflect broader systemic disturbances rather than direct neuropathic mechanisms. This limitation highlights the need to shift the focus from isolated miRNA biomarkers to a more comprehensive analysis of the circulating MiRnome, the global miRNA expression profile, which reflects coordinated regulatory networks involved in gene expression control rather than the activity of individual miRNAs. Such an approach could offer a more nuanced understanding of the molecular landscape of DN, capturing the complex interplay of inflammation, oxidative stress, and metabolic dysregulation that characterizes the disease [81,82]. By embracing the MiRnome as a diagnostic and therapeutic framework, researchers and clinicians can better identify meaningful patterns and targets that transcend the constraints of single-marker specificity. Moreover, a MiRnome panel could allow for stratification of patients based on disease stage, severity, and response to treatment. This approach may improve specificity over single miRNA markers by capturing the broader pathophysiological context. Blood-based MiRNome analysis (e.g., from serum, plasma, or leukocytes) offers a minimally invasive tool for monitoring disease progression and therapeutic efficacy.

Although several biomarkers are already well established and widely recognized in both scientific literature and clinical practice, improving their sensitivity and pathophysiological specificity remains a substantial need. In this context, the integration of advanced in vitro models into biomarker research represents a strategic approach to bridge mechanistic insights with translational applicability. By enabling the controlled investigation of disease-relevant molecular pathways and cellular responses, these systems provide a robust platform for the identification, functional characterization, and preliminary validation of novel biomarker candidates, thereby contributing to the refinement and potential expansion of current diagnostic and prognostic frameworks. For this reason, the following section addresses the potential of neuronal and glial in vitro models as translational platforms, highlighting how these systems can be leveraged to investigate biomarker dynamics, validate candidate molecules in a mechanistic context, and ultimately strengthen the bridge between experimental findings and clinical application.

## 5. In Vitro Methods to Study Glial and Axonal Damage in Diabetic Peripheral Neuropathy

**Figure 3:**
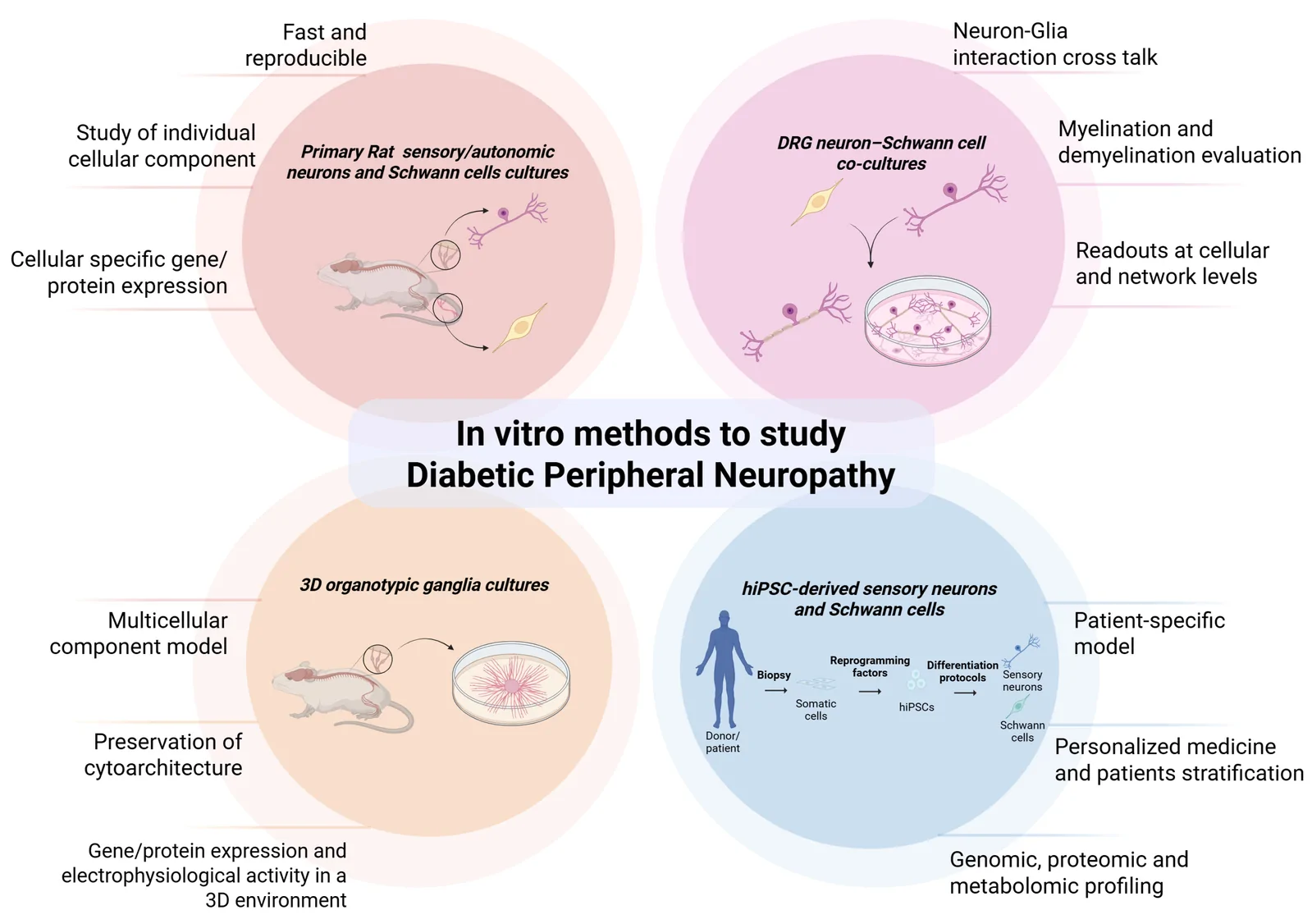
In vitro methods for studying diabetic peripheral neuropathy. Schematic overview of neuronal and glial in vitro models useful in diabetic neuropathy studies. Created in BioRender. Fregnan, F. (2026) https://BioRender.com/yf3tarj.

Because DN affects both neuronal and glial components of the peripheral nerve, in vitro neuronal and glial models offer valuable tools to investigate the early pathogenic events induced by diabetic conditions. Elucidating the distinct kinetics of degeneration and regeneration following injury, and how these processes are altered in the context of DN, is crucial for understanding disease pathophysiology and intervention strategies [83]. In vitro models allow for precise control of glucose levels and enable real-time assessment of oxidative stress, mitochondrial dysfunction, neuroinflammatory signaling, and impaired axonal transport in a diabetic environment. By integrating advanced morphological, molecular, and functional methodologies, including microfluidic and electrophysiological analyses, they allow for detailed exploration of hyperglycemia-induced neurodegenerative processes and facilitate the identification of potential biomarkers linked to disease onset, progression, and therapeutic response. This understanding is essential for uncovering novel therapeutic targets and for studying neuroprotective interventions aimed at preventing or mitigating the progression of DN. This in vitro modeling approach allows candidate biomarkers to be evaluated with respect to their cellular origin, temporal appearance, relationship to disease mechanisms, and responsiveness to interventions before they are tested in complex biological fluids or clinical cohorts. For instance, NfL, already recognized as a marker of axonal injury in clinical and experimental studies [47,48,49], could be investigated in neuronal or neuron–glia in vitro models to determine its cellular origin, release kinetics, and relationship to axonal degeneration. Similarly, PRPH, which has been reported to increase in diabetic sensory and sympathetic neurons of experimental models [52,53], could be investigated in hyperglycemic neuronal in vitro models to determine whether changes in its expression and localization are linked to neurite fragmentation, impaired axonal outgrowth, or altered neuronal viability. This would help establish PRPH as a model-supported biomarker of diabetes-related axonal injury before its evaluation in patient samples. Thus, in vitro studies can serve as a translational bridge between mechanistic research and clinical biomarker development, supporting their identification, prioritization, and preliminary functional validation. However, their analytical, biological, and clinical validation must subsequently be confirmed in human samples and prospective clinical studies. For these reasons, in the following sections, several neuronal and glial in vitro models are described and considered for the study of DN in different suitable experimental conditions (Figure 3).

### 5.1. Primary Cultures of Sensory Neurons and Schwann Cells

Primary cell cultures can closely mimic the in vivo physiology of the animal, which makes them more representative than immortalized cell lines. However, there are species differences between murine and human cells, so translational relevance should be carefully evaluated. Data obtained from rat primary cells may not fully predict human outcomes. High variability and restricted scalability are coupled with ethical concerns about the use of animals. However, to mimic and study DN, it is possible to take advantage of at least two main cell types: primary DRG neurons and SCs. DRG sensory neurons are widely used as an in vitro model to investigate peripheral sensory neuron biology and to study key mechanisms of high-glucose-induced nerve damage relevant to DN. Indeed, in vitro exposure to high-glucose conditions recapitulates key features of the diabetic phenotype, including reduced neurite outgrowth, mitochondrial fragmentation, and increased expression of apoptotic markers, consistent with the DRG-specific oxidative stress reported in diabetic pain models [84,85]. These cultures therefore represent a tractable platform for dissecting the mechanisms underlying sensory deficits and for evaluating potential neuroprotective strategies.

Clear morphological and molecular changes under diabetic conditions make these cells essential for the reliable quantification of neurodegeneration [86]. At the same time, primary neurons are post-mitotic and fragile, which limits long-term studies and repeated manipulations.

Another in vitro model consists of primary SCs. When exposed to diabetic-mimetic conditions, these cells exhibit down-regulation of key myelin proteins (MBP, P0), increased reactive oxygen species production, and a shift toward a pro-inflammatory cytokine profile, consistent with oxidative stress-induced apoptosis and inflammatory dysregulation [87]. However, similarly to primary DRG neurons, primary SCs tend to lose the myelinating phenotype after only a few passages, which often hinders long-term studies.

### 5.2. Co-Culture Systems

By culturing DRG neurons together with SCs, it is possible to closely recreate the physiological bidirectional communication that exists between neurons and glial cells in peripheral nerves. This neuronal–glial crosstalk is essential for maintaining axonal integrity, myelin formation, and neuronal survival and is profoundly disrupted under hyperglycemic conditions, as observed in DN. Within such co-culture systems, elevated glucose levels impair neurotrophic signaling, particularly through reduced production of, or responsiveness to, growth factors such as NGF and BDNF. These factors are essential for neuronal survival and axonal regeneration, and their dysregulation represents one of the earliest molecular consequences of diabetic injury [60,88].

Furthermore, hyperglycemia can lead to aberrant axon–myelin communication, affecting the ability of SCs to properly ensheath or remyelinate axons and resulting in functional deficits that mimic in vivo pathology [89]. At the same time, metabolic stress can trigger the activation of pro-inflammatory pathways within both cell types, such as NF-κB or MAPK signaling, leading to the release of cytokines and reactive oxygen species that exacerbate cellular dysfunction and neurodegeneration.

The use of conditioned medium experiments, in which the culture medium from neurons is applied to SCs (or vice versa), represents an elegant strategy for dissecting the molecular dialogue between the two populations [90]. This approach allows researchers to identify secreted mediators, such as growth factors, cytokines, or extracellular vesicles, that contribute to either neuroprotection or injury [91]. Altogether, the DRG neuron–SC co-culture model provides a powerful and physiologically relevant system for studying how hyperglycemia disturbs neuron–glia communication and testing potential therapeutic strategies aimed at restoring this balance.

### 5.3. 3D Organotypic Cultures of Sensory and Autonomic Ganglia

Sensory and autonomic explants provide a versatile “middle-ground” platform that captures many of the structural and cellular complexities of an intact peripheral nerve while retaining the experimentally tractability of an in vitro system [92,93]. In a three-dimensional organotypic culture, the native ganglionic architecture, satellite glial cells, and long-range axonal projections are preserved, which allows researchers to monitor axonal projections and degeneration and neuroinflammatory signaling under diabetic-mimicking conditions. Under controlled experimental conditions, explant ganglia can be exposed to sustained hyperglycemia, elevated levels of AGEs, or serum from diabetic animals or patients. Unlike dissociated neuron cultures, which enable investigation of neuron-intrinsic effects of hyperglycemia, 3D organotypic cultures preserve the native multicellular environment [94], including satellite glial cells, SCs, and extracellular matrix, and enable the study of tissue-level phenomena such as glial activation, impaired axonal fasciculation, disrupted neuron–glia interactions, and inflammation, which are critical features of DN. Consequently, dissociated cultures are ideal for mechanistic, cell-autonomous studies, whereas explants better model the complex, physiological interplay underlying diabetic nerve injury and regeneration.

### 5.4. Human-Induced Pluripotent Stem Cell (hiPSC) Cultures

In recent years, increasing attention has been devoted to the development of differentiation protocols for hiPSCs [95]. By reprogramming patients’ skin fibroblasts into pluripotent cell lines and then differentiating them into different cell types, these platforms enable the generation of personalized models that capture individual genetic backgrounds and disease phenotypes. The use of human-derived systems minimizes interspecies differences, thereby supporting the development of more predictive experimental models and potentially reducing the high attrition rates observed in clinical trials. However, the use of hiPSC-derived cells is limited by complex, time-consuming differentiation protocols and incomplete functional maturity, which constrain their direct translational relevance to adult human models. Despite these limitations, hiPSC technology offers a powerful and flexible platform for modeling DN in a human-relevant context. Indeed, by directing hiPSCs toward sensory neuron and glial lineages, it is possible to generate peripheral nerve components in vitro, such as nociceptors, mechanosensory neurons, and SCs [96,97,98,99,100]. When these cellular types are exposed to hyperglycemic or oxidative stress conditions in vitro, they exhibit hallmark features of DN, such as reduced neurite outgrowth, altered ion channel expression, impaired axonal transport, and increased apoptosis, closely recapitulating disease phenotypes observed in patients [101].

Moreover, co-culturing hiPSC-derived sensory neurons with SCs enables investigation of myelination defects, disrupted neurotrophic signaling, and inflammatory responses that contribute to peripheral nerve degeneration. Through this approach, bidirectional neuron–SC interactions can be investigated in a fully human model, which can provide mechanistic insights into the processes driving demyelination and axonal loss in DN [98,99,100,102].

Notably, hiPSC-derived models strongly support personalized medicine approaches [103]. The use of hiPSCs generated from patients with DM allows for the investigation of individual susceptibility to neuropathy, the identification of genetic or epigenetic modifiers, and the screening of potential neuroprotective compounds in a patient-specific context [104]. Importantly, the integration of hiPSCs with advanced techniques such as organoid cultures, microfluidic “nerve-on-a-chip” platforms, and three-dimensional co-culture systems further enhances physiological relevance by recapitulating the complex microenvironment of peripheral nerves [105,106]. These innovations make hiPSC-based systems a promising tool not only for understanding DN pathogenesis but also for testing novel therapeutic strategies aimed at preventing or reversing nerve damage in diabetic patients.

## 6. Experimental Diabetic Conditions

To model the metabolic and ischemic stressors underlying DN, in vitro systems commonly employ high-glucose exposure and oxygen deprivation. These complementary paradigms recapitulate key pathological features of DM and are described in detail in the following sections.

First, high-glucose exposure is a widely employed strategy to recapitulate key pathological features of DN in vitro. These conditions, typically ranging from 25 to 150 mM glucose in the literature, induce oxidative stress, mitochondrial dysfunction, impaired neurite outgrowth, and altered gene expression profiles associated with inflammation and neurodegeneration [87,107]. The design of an in vitro diabetic neuropathy model requires careful separation of glucose-dependent effects from non-specific osmotic, nutritional, and culture-related stress. A high-glucose condition should be paired with an equimolar mannitol control to distinguish glucose-specific effects from osmotic stress, and glucose concentrations should be reported as final measured concentrations rather than only nominal media values [108]. When combined with complementary stressors such as hypoxia or AGEs, high-glucose treatment enhances the translational relevance of the model.

Additionally, hypoxic conditions are frequently employed to simulate the ischemic component of DN in vitro. Two complementary methods are commonly used to induce oxygen deprivation: chemical hypoxia via cobalt chloride (CoCl_2_), which stabilizes hypoxia-inducible factor 1-alpha (HIF-1α) and mimics cellular responses to low oxygen; and physical hypoxia using a hypoxic chamber, where oxygen levels are precisely reduced, typically to 1–5% O_2_, to replicate chronic tissue hypoxia [109,110]. Both approaches trigger metabolic reprogramming, oxidative stress, and altered expression of genes involved in angiogenesis, inflammation, and neurodegeneration. When combined with hyperglycemic stress, hypoxia enhances the pathophysiological relevance of the in vitro DN model, enabling the study of synergistic damage mechanisms and the testing of neuroprotective interventions.

The temporal structure of the experiment is equally important. Short exposures may reveal early metabolic and signaling events, including ROS generation, mitochondrial dysfunction, altered calcium handling, NF-κB activation, and changes in trophic-factor expression, whereas prolonged exposures may result in neurite retraction, axonal fragmentation, neuronal apoptosis, SC dedifferentiation, impaired myelin gene expression, and reduced support for axonal regeneration [41,111,112,113,114,115,116,117,118]. Considering several time points can therefore distinguish initiating mechanisms from secondary dependent injury responses and help determine whether a candidate biomarker precedes, parallels, or follows morphological damage. For neurons, the experimental design should include assessment of neurite length and branching, axonal integrity, neuronal viability, mitochondrial membrane potential, and apoptosis. For SC models, relevant outcomes include cell viability; proliferation; migration; myelin-associated proteins such as MBP, MAG, and P0; repair-related markers such as p75NTR; and secretion of inflammatory or neurotrophic mediators. In neuron–glial co-cultures, these measurements should be combined with cell-type-specific immunostaining or molecular analysis so that a change in a secreted biomarker can be directly assigned to neuronal or glial injury rather than to the mixed culture as a whole.

Because DN develops in the context of interacting metabolic and vascular abnormalities, glucose alone may not reproduce the full disease phenotype. Where biologically justified, models can incorporate additional stressors such as hypoxia or hypoxia–reoxygenation, AGEs, palmitate or other lipid stressors, inflammatory cytokines, altered insulin signaling, or serum/plasma from individuals with diabetes [119,120,121,122,123]. These conditions should be introduced in a factorial or otherwise controlled design rather than combined indiscriminately, because each added stressor increases interpretive complexity. Indeed, a model containing high glucose, hypoxia, and inflammatory cytokines may be useful for testing convergent injury pathways, but it cannot establish which component is responsible for a specific biomarker change unless the individual conditions and relevant combination controls are included.

Model validity also depends on experimental quality and reproducibility. Passage number, cell density, substrate coating, serum concentration, medium changes, and exposure duration should be standardized and reported [124]. Appropriate technical replicates and independent biological experiments are required, and investigators should distinguish biological replication from repeated measurements within the same culture. Cell viability and osmolality should be monitored in parallel with injury readouts to exclude the possibility that an apparent increase in a biomarker simply reflects widespread non-specific cell death. Finally, rescue experiments using antioxidants, RAGE-pathway modulators, mitochondrial protectants, or other mechanistically selected interventions can help determine whether a candidate biomarker tracks a biologically relevant injury pathway and changes in response to partial protection. Such experiments do not establish clinical validity, but they strengthen the mechanistic rationale for prioritizing biomarkers for subsequent measurement in human samples [112,125,126,127,128].

## 7. Methods to Assess Cell Impairment

Cellular dysfunction in nerve damage in vitro models can be evaluated using complementary morphological, molecular, biochemical, and functional approaches. The methods outlined below enable the assessment of neuronal and glial alterations, from structural degeneration to molecular and electrophysiological deficits, under diabetic conditions.

### 7.1. Morphological Analysis

Beyond the use of optical microscopy and classical morphological techniques, such as bright-field and phase-contrast microscopy, as well as conventional histological stains (hematoxylin and eosin, Nissl staining), which allow for the evaluation of cell morphology in vitro, immunofluorescence represents a highly valuable approach. Using fluorescently labeled antibodies, it is possible to visualize and quantify the expression and spatial distribution of key structural and functional proteins, which can provide insight into cellular health and neuron–glial interactions. Immunofluorescence staining combined with confocal microscopy represents a powerful approach for exploring the morphological alterations of neuronal and glial cells associated with DN. In neuronal cultures, markers such as βIII-tubulin, peripherin, and Nfl are commonly used to assess neurite integrity, axonal branching, and cytoskeletal organization, while synapsin-1 or MAP2 can reveal changes in synaptic structure and dendritic morphology. In parallel, SC phenotype and function can be characterized by markers like S100β, myelin basic protein (MBP), and P0, which indicate myelination status and glial differentiation. Under hyperglycemic conditions, immunofluorescence imaging can uncover neurite retraction, demyelination, and abnormal protein localization, which reflect the degenerative processes typical of DN. The use of confocal microscopy enables the high-resolution, three-dimensional reconstruction of cellular architecture, allowing for precise analysis of axon–SC interactions, myelin sheath integrity, and subcellular protein colocalization [129].

By combining immunofluorescence and confocal microscopy with image analysis software, it is possible to quantitatively assess important features of the in vitro cultures. For instance, neurite outgrowth is a critical readout for evaluating neuronal health and regenerative capacity in in vitro models of DN. Skeletonization techniques, often implemented using image analysis software like ImageJ or CellProfiler, transform fluorescently labeled neurites into simplified, binary representations, enabling precise morphometric quantification of parameters like total neurite length, number of branches, and Sholl intersections [130]. These metrics provide insights into axonal degeneration, impaired growth, and cytoskeletal remodeling, which are hallmark features of DN.

The ImageJ Skeletonize plugin reduces a binary image to a single-pixel skeleton, from which total branch length and branch point number can be extracted quickly. However, it relies on a preceding threshold step, lacks branch-order discrimination, and can produce fragmented skeletons in dense networks, which makes it less suitable for explant cultures where neurites overlap extensively. Conversely, Neurite-J’s ability to distinguish primary-through-tertiary branches and to generate detailed masks makes it the preferred tool for complex explant preparations. The validation of Neurite-J against manual tracing demonstrated a high correlation, confirming the reliability of these automated metrics for assessing neurite dynamics in three-dimensional organotypic cultures [94].

Finally, NeurphologyJ is another ImageJ that focuses on morphological descriptors such as soma size, neurite thickness, curvature, and branching complexity. It excels at quantifying fine-scale shape parameters and can be applied to both explant and dissociated cultures after appropriate segmentation. However, because NeurphologyJ does not directly calculate cumulative neurite length or branch-order hierarchies, it is best used in conjunction with Neurite-J (for explants) or Skeletonize (for dissociated cells) when detailed morphological profiling of individual neurons is required [131].

In practice, a workflow that combines Neurite-J for global outgrowth metrics, Skeletonize for rapid length estimation in simple cultures, and NeurphologyJ for detailed shape analysis offers the most versatile strategy for evaluating DRG experimental models.

### 7.2. Biochemical and Molecular Investigation

Biochemical and molecular analyses are indispensable for dissecting the complex molecular mechanisms underlying DN. Real-time quantitative PCR (RT-qPCR) is a powerful tool for investigating transcriptional alterations across multiple pathogenic pathways. One key focus is the neurotrophic signaling pathway, where reduced expression of NGF, BDNF, and their receptors (TrkA, TrkB, and p75NTR) reflects impaired neuronal support and survival. Altered regulation of PI3K–Akt and MAPK–ERK downstream targets, such as AKT1, ERK1/2, and CREB, can be analyzed to evaluate how trophic deprivation compromises neuronal maintenance and regeneration [132,133]. Regarding oxidative stress and mitochondrial dysfunction pathways, RT-qPCR quantification of genes such as SOD1, CAT, GPX1, Nrf2, and HMOX1 provides insight into the antioxidant defense capacity of neurons and SCs under chronic hyperglycemic stress. Simultaneous assessment of NOX4, iNOS, and COX2 expression can reveal the activation of pro-oxidant and inflammatory cascades. In addition, the inflammatory signaling network can be explored by measuring transcriptional levels of TNF-α, IL-1β, IL-6, and MCP-1, alongside components of the NF-κB pathway (RELA, IKBα, and TLR4), to delineate the molecular mediators driving neuroinflammation. These changes often correlate with SC activation and neuronal damage. Moreover, apoptotic and autophagic pathways can be examined by profiling BAX, BCL2, CASP3, CASP9, Beclin-1, and LC3, providing molecular evidence of cell death or survival responses triggered by hyperglycemic conditions. In parallel, RT-qPCR in SCs can target genes involved in myelination and glial maintenance, such as MPZ (P0), MBP, Krox20, Sox10, and ErbB2/3, which are often dysregulated during demyelination in DN [134].

Complementary protein quantification assays, such as Western blotting or colorimetric/fluorometric protein assays, enable the assessment of corresponding protein expression and post-translational modifications, providing a direct link between transcriptional changes and cellular phenotype.

Enzyme-linked immunosorbent assays (ELISAs) further enhance sensitivity and specificity for quantifying secreted growth factors, cytokines, and metabolic markers within culture media, offering a detailed view of the extracellular environment. In addition, secretome profiling, through multiplex ELISA arrays or mass spectrometry-based proteomics, can comprehensively characterize the spectrum of molecules released by neurons and SCs, identifying potential mediators of axonal degeneration, inflammation, or neuroprotection. This method involves capturing target proteins with immobilized antibodies, followed by detection with enzyme-conjugated secondary antibodies and a colorimetric or fluorescent readout. By comparing secretome profiles under normoglycemic, hyperglycemic, and hypoxic conditions, researchers can identify dysregulated signaling pathways, such as pro-inflammatory cascades (e.g., IL-6, TNF-α), impaired neurotrophic support (e.g., NGF, BDNF), or oxidative imbalance (e.g., 8-OHdG, H_2_O_2_). ELISA-based secretome analysis thus provides a functional readout of cellular stress responses and intercellular communication, complementing morphological and electrophysiological assessments in human-relevant DN models [135].

### 7.3. Electrophysiological Recordings

Electrophysiological measurements are essential for assessing functional integrity and excitability in in vitro models of DN. Two complementary techniques are commonly employed: patch clamp recordings and multi-electrode array (MEA) systems. Patch clamp offers high-resolution, single-cell analysis of ion channel activity, membrane potential, and action potential dynamics, which makes it ideal for detailed mechanistic studies of neuronal dysfunction under hyperglycemic or hypoxic stress. By contrast, MEA systems enable non-invasive, long-term monitoring of spontaneous and evoked electrical activity across neuronal networks, providing population-level insights into connectivity, firing patterns, and drug responsiveness. Together, these methods allow researchers to capture both cellular and network-level electrophysiological alterations, enhancing the translational relevance of in vitro DN models and supporting the development of targeted neuroprotective therapies [136,137].

### 7.4. Microfluidic Platforms

Microfluidic platforms are miniaturized, chip-based systems designed to manipulate small volumes of fluids with high precision, which makes them ideal for creating controlled cellular microenvironments in vitro. These devices typically consist of networks of microchannels, allowing for the spatial segregation of different cell types, the establishment of chemical gradients, and the application of localized stimuli. Their architecture supports dynamic perfusion, long-term culture, and real-time imaging while maintaining physiological relevance at microscale resolution. In the context of neurobiology and disease modeling, microfluidic platforms enable the isolation of axons from neuronal somas, the study of directional growth, and the investigation of cell–cell interactions under pathophysiological conditions. This level of control enhances the physiological relevance of DN models, enabling the study of axonal degeneration, myelination defects, and neuroinflammatory responses under diabetic conditions. Moreover, microfluidic platforms are compatible with live cell imaging, immunostaining, and even electrophysiological readouts, which makes them ideal for high-content screening and mechanistic investigations in human-relevant systems [138].

## 8. Discussion

DN is a complex and progressive complication involving both neuronal and glial components of the peripheral nervous system. This review provides an overview of DN pathophysiology, the disease and its wide target, with a specific focus on axonal and glial damage. The societal impact is profound: DN drives increased morbidity, loss of productivity, and elevated mortality, while current clinical management is limited to symptom control and risk-factor modification; this underscores an urgent need for reliable biomarkers to guide early intervention [139].

Prompt detection is crucial for initiating timely therapeutic interventions aimed at slowing or preventing nerve damage progression, ultimately improving patient outcomes.

Despite advances in diagnostic techniques, a major limitation persists: most tools detect neuropathy after structural damage has already occurred. There is a clear diagnostic gap in identifying early-stage, subclinical neuropathy, when patients are still asymptomatic but nerve damage has begun. In the context of DN, neuronal injury biomarkers reflect ongoing nerve damage at a molecular level, often before clinical symptoms or electrophysiological abnormalities are evident.

For instance, elevated NfL levels have been associated with early axonal damage in diabetic patients even before nerve conduction abnormalities are detectable [140]. Early diagnosis can help clinicians stratify patients by risk and monitor disease progression more precisely. Biomarkers can assist in personalizing treatment strategies by identifying patients who are most likely to benefit from neuroprotective or disease-modifying therapies. Tracking changes in biomarker levels may also serve as an objective measure of therapeutic response, facilitating adjustments in management plans [141]. For example, reductions in inflammatory cytokines or oxidative stress markers could reflect effective glycemic control or antioxidant therapy.

Emerging evidence supports the potential of protein-based biomarkers, such as Nfl and NSE, alongside inflammatory and metabolic markers, in detecting early neuronal damage in diabetic patients [48,64,67,75,140,142]. These biomarkers could revolutionize the diagnostic landscape by enabling earlier diagnosis, personalized treatment, and improved monitoring of disease progression.

Despite promising research, integrating neuronal injury biomarkers into routine clinical practice requires overcoming several challenges, including assay standardization, cost-effectiveness, and the establishment of clinically relevant threshold values. Large-scale, longitudinal studies are needed to validate biomarkers and develop guidelines for their interpretation and use [143].

At the same time, experimental models that mirror pathogenic changes seen in patients could become important discovering tools, enabling the study and identification of new, reliable biomarkers that bridge the clinical and research fields. In contrast to in vivo models, in vitro systems provide a controlled environment in which a specific pathogenic stimulus, including high glucose or hypoxic stress, can be selectively applied to a defined cell type, thus enabling a more refined understanding of the cellular events driving diabetic neuropathy. The in vitro experimental systems discussed in this review provide a tightly controlled setting for investigating hyperglycemia-induced neurodegeneration, enabling the study of pathogenic pathways that are not yet fully elucidated. Examples include rat primary cultures of DRG neurons and SCs, which allow for detailed analysis of hyperglycemia-induced neurodegeneration, while co-culture systems reproduce the neuron–glia interactions and enable assessment of the molecular pathways underlying axonal degeneration, glial cell damage, and demyelination in DN [84,88,104,110,137]. As a step further, organotypic 3D cultures preserve native tissue architecture and neuron–glia interactions, providing a bridge between isolated cell assays and whole-animal models [92,94]. Compared with conventional monolayer cultures, the organotypic format better reproduces extracellular-matrix cues and cell–cell contacts that modulate neuronal excitability and metabolic stress, thereby yielding phenotypes that more closely resemble the progressive loss of sensory fibers seen in DN. Notably, these explants encompass both somatic and autonomic representations of the peripheral nervous system: DRGs serve as a sensory (somatic) model, whereas three-dimensional cultures derived from vagal ganglia and the superior cervical ganglia provide an autonomic counterpart; this makes this approach particularly suitable for the in vitro investigation of both sensory and autonomic forms of DN.

Recently, increasing attention has been directed toward the use of hiPSC-derived neurons and glia as a human-relevant platform that not only overcomes interspecies differences but also retains patient-specific genetic backgrounds and epigenetic signatures [95]. This dual advantage enables more accurate modeling of disease susceptibility and drug responses, promoting the discovery of clinically relevant biomarkers and advancing the principles of personalized medicine. Combined with advanced morphological, molecular, and functional assays, these models offer powerful tools for elucidating DN pathogenesis and screening potential neuroprotective therapies in a controlled yet physiologically relevant environment. Beyond their role in elucidating DN mechanisms, these in vitro systems also offer a valuable platform for exploring how circulating factors influence neuronal and glial integrity, thereby supporting the discovery and preliminary testing of potential circulating biomarkers [96,97,99,100].

## 9. Conclusions

In conclusion, advanced in vitro models that mimic the diabetic in vivo environment remain a significant challenge, yet they are crucial for both basic and translational research. Such models not only allow precise dissection of the molecular and cellular pathways driving DN, deepening the understanding of its pathophysiology, but also provide an experimental approach to assess and validate the relevance of biomarkers in physiological and diabetic contexts. A model-based strategy can accelerate this process: controlled neuronal, Schwann cell, and neuron–glia in vitro systems allow candidate biomarkers to be discovered under defined diabetic conditions, linked to specific cellular and functional changes, and prioritized before evaluation in patient samples. By bridging experimental insights with translational applications, these refined models hold the potential to accelerate the development of targeted therapeutic strategies. However, translating these promising biomarkers from research to clinical practice requires standardized assays, robust validation in diverse populations, and clear clinical guidelines. Future studies should focus on longitudinal evaluations and the integration of multimodal biomarker panels to enhance sensitivity and specificity. Ultimately, incorporating early neuronal injury biomarkers into clinical workflows holds promise for improving outcomes for patients with DN by facilitating timely interventions that prevent irreversible nerve damage.

## Figures and Tables

**Table 1:** Fiber involvement in different types of neuropathy.

	Autonomic Neuropathy	Somatic Neuropathy
Type of neuropathy	• CAN • Gastroenteropathy • Sudomotor dysfunction • Urogenital neuropathies • Ocular neuropathy	• Distal sensorimotor peripheral neuropathy
Fiber type	• Aδ (thin myelinated) • C (unmyelinated)	• Aδ (thin myelinated) • C (unmyelinated) • Aα and Aβ (thick myelinated)
Clinical onset	Early and insidious	Late and progressive

## Data Availability

No new data were created or analyzed in this study. Data sharing is not applicable to this article.

## Abbreviations

The following abbreviations are used in this manuscript:

AGE	advanced glycation end-products
BAX	Bcl-2-associated X protein
BCL2	B-cell lymphoma 2
BDNF	brain-derived neurotrophic factor
CAN	Cardiac Autonomic Neuropathy
CASP3/9	caspase 3/9, apoptosis-related cysteine peptidase
CAT	catalase
CGRP	calcitonin gene-related peptide
cmRNA	circulating mRNA
COX2	cytochrome c oxidase subunit 2
DM	diabetes mellitus
DN	diabetic peripheral neuropathy
eNOS	endothelial nitric oxide synthase
ELISA	enzyme-linked immunosorbent assay
ERBB2/3	Erb-B2 receptor tyrosine kinase 3/3
FGFR3	fibroblast growth factor receptor 3
GBS	Guillain–Barré syndrome
GFAP	glial fibrillary acidic protein
GPX1	glutathione peroxidase 1
HIF-1α	hypoxia-inducible factor 1-alpha
hiPSC	human-induced pluripotent stem cell
HMOX1	heme oxygenase 1
IENFD	Intraepidermal Nerve Fiber Density
IKBα	Nuclear factor of kappa light polypeptide gene enhancer in B-cells inhibitor, alpha
IL-1β	interleukin 1 beta
IL-6	interleukin 6
iNOS	inducible nitric oxide synthase
KROX2	(EGR2) early growth response 2
LC3	microtubule-associated protein 1 light chain 3
MAG	myelin-associated glycoprotein
MAPK	mitogen-activated protein kinase
MBP	myelin basic protein
MCP-1	monocyte chemoattractant protein 1
MEA	multi-electrode array
miRNA	microRNA
MPZ	myelin protein zero
mTORC1	mechanistic target of rapamycin complex 1
NADPH	nicotinamide adenine dinucleotide phosphate
NF-κB	nuclear factor-kappa B
NfL	Neurofilament Light Chain
NGF	nerve growth factor
NOX4	NADPH oxidase 4
Nrf2	nuclear factor erythroid 2–related factor 2
NSE	Neuron-Specific Enolase
P0	protein zero
PKC	protein kinase C
PRPH	peripherin
RAGE	advanced glycation end-products receptor
RELA	v-rel avian reticuloendotheliosis viral oncogene homolog A
ROS	reactive oxygen species
SC	Schwann cell
SOD1	superoxide dismutase 1
SOX10	SRY-box transcription factor 10
SP	substance P
T1DM	type 1 diabetes mellitus
T2DM	type 2 diabetes mellitus
TLR4	toll-like receptor 4
TNF-α	tumor necrosis factor alpha
VEGF	vascular endothelial growth factor
